# Supplementary material for: Mortality over time among COVID-19 patients hospitalized during the first surge of the pandemic: A large cohort study
Source: PLoS One. 2022 Sep 28;17(9):e0275212. doi: 10.1371/journal.pone.0275212 (PMC9518866; doi:10.1371/journal.pone.0275212)
Supplement: S1 File — (DOCX) [file pone.0275212.s001.docx]

**Supporting Information**

Mortality over time among COVID-19 patients hospitalized during the first surge of the

pandemic: a large cohort study

**Author names and affiliations:**

Izabel Marcilio^1^

Felippe Lazar Neto^2^

Andre Lazzeri Cortez^1^

Anna Miethke-Morais^1^

Hillegonda Maria Dutilh Novaes^3^

Heraldo Possolo de Sousa^2^

Carlos R. R. Carvalho^4^

Anna Sara Shafferman Levin^1^

Juliana Carvalho Ferreira^4^

Nelson Gouveia^3^

HCFMUSP COVID-19 Study Group*^1^

^1^ Hospital das Clinicas da Faculdade de Medicina da Universidade de Sao Paulo (HCFMUSP) - Av. Dr. Enéas Carvalho de Aguiar, 255. 05403-000, São Paulo, Brazil

^2^ Emergency Department, Hospital das Clínicas da Faculdade de Medicina da Universidade de Sao Paulo - Av. Dr. Enéas Carvalho de Aguiar, 255. 05403-000 São Paulo, Brazil

^3^ Faculdade de Medicina, Universidade de Sao Paulo - Av. Dr. Arnaldo, 455. 01246-903, São Paulo, Brazil

^4^ Divisao de Pneumologia, Instituto Do Coracao, Hospital das Clinicas da Faculdade de Medicina da Universidade de Sao Paulo (HCFMUSP) - Av. Dr. Eneas Carvalho de Aguiar, 44. 05403-000, Sao Paulo, Brazil

** HCFMUSP COVID-19 Study Group: Tarcisio E.P. Barros-Filho, Eloisa Bonfa, Edivaldo M. Utiyama, Aluisio C. Segurado, Beatriz Perondi, Amanda C. Montal, Leila Harima, Solange R.G. Fusco, Marjorie F. Silva, Marcelo C. Rocha, Izabel Cristina Rios, Fabiane Yumi Ogihara Kawano, Maria Amélia de Jesus, Esper Kallas, Maria Cristina Peres Braido Francisco, Carolina Mendes do Carmo, Clarice Tanaka, Maura Salaroli Oliveira, Thaís Guimarães, Carolina dos Santos Lázari, Marcello M.C. Magri, Julio F.M. Marchini, Alberto José da Silva Duarte, Ester C. Sabino, Silvia Figueiredo Costa*

**S1 Fig. Admissions according to month in 2020**

**
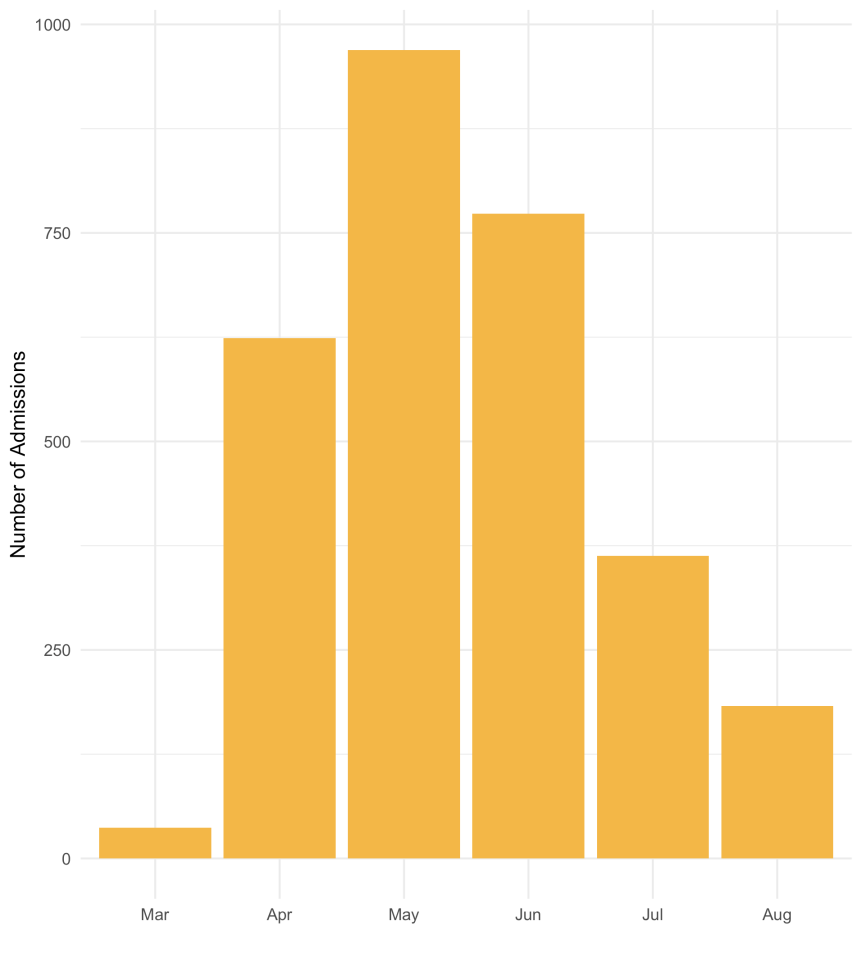
**

**Footnote:** Bars represent the number of admissions by month in 2020 during the study period

**S2 Fig. Comparison of survivors and nonsurvivors**

**
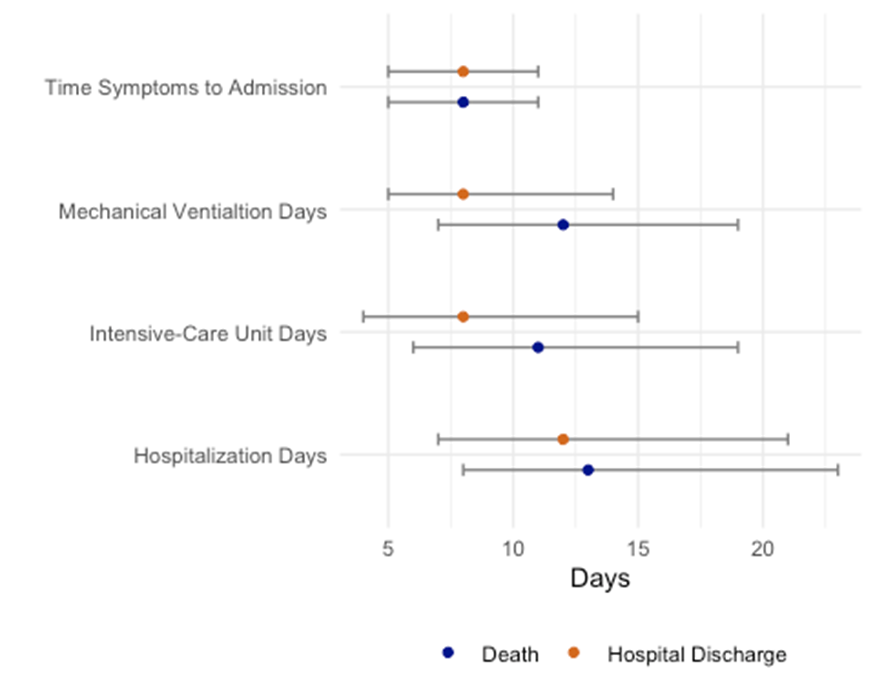
**

**Footnote:** Comparison of the median duration the several important characteristics between survivors (blue dots) and nonsurvivors (orange dots). Bars represent the 25%-75% interquartile percentiles

**S3 Fig. Use of corticosteroids over time**


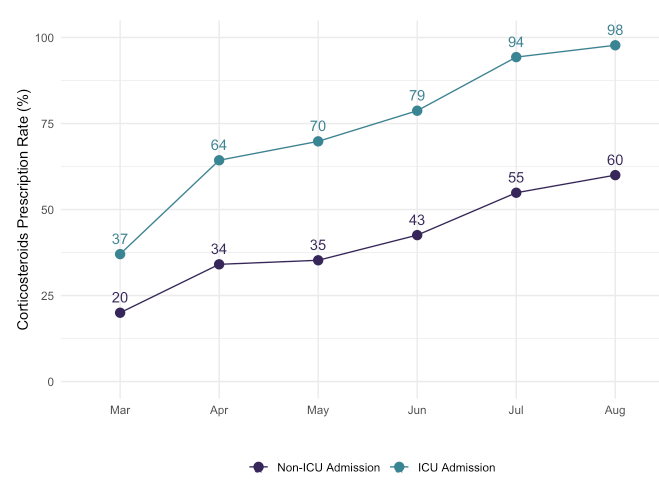


**Footnote:** Percent of patients admitted to the ICU (green dots) or ward (dark blue dots) receiving systemic corticosteroids over time

|  | **Hospital Discharge (N=1807)** | **Death (N=956)** | **Missing %** |
| --- | --- | --- | --- |
| **Age,** *Median (IQR)* | 56.8 (43.5, 67.8) | 67.3 (58.7, 74.9) | 0,00% |
| **Age Groups (Years)** |  |  | 0,00% |
| 18-49 | 660 (36.5%) | 117 (12.2%) |  |
| 50-59 | 366 (20.3%) | 146 (15.3%) |  |
| 60-69 | 408 (22.6%) | 299 (31.3%) |  |
| 70-79 | 258 (14.3%) | 255 (26.7%) |  |
| 80+ | 115 (6.4%) | 139 (14.5%) |  |
| **Male Sex** | 956 (52.9%) | 601 (62.9%) | 0,00% |
| **Admission Place** |  |  | 0,00% |
| General Wards | 1109 (61.4%) | 192 (20.1%) |  |
| Intensive Care Unit | 688 (38.1%) | 761 (79.6%) |  |
| Emergency | 10 (0.6%) | 3 (0.3%) |  |
| **Health Care Professional** | 67 (8.6%) | 4 (1.0%) | 57,15% |
| **Smoking** |  |  |  |
| Current Smoker | 91 (5.0%) | 83 (8.7%) | 0,18% |
| Previous Smoker | 361 (20.1%) | 213 (22.3%) | 0,00% |
| **Pregnancy** | 128 (15.0%) | 5 (1.4%) | 0,00% |
| **Syptoms Onset,** *Median (IQR)* | 7.0 (5.0, 11.0) | 7.0 (4.0, 11.0) | 0,07% |
| **CCI,** *Median (IQR)* | 2.0 (1.0, 2.0) | 2.0 (1.0, 3.0) | 4,42% |
| **Comorbidities** |  |  |  |
| Hypertension | 981 (54.3%) | 622 (65.1%) | 0,07% |
| Diabetes | 617 (34.2%) | 420 (43.9%) | 0,07% |
| Cardiovascular Disease | 294 (16.3%) | 193 (20.3%) | 0,22% |
| Obesity | 285 (15.8%) | 91 (9.5%) | 0,00% |
| Cancer (Solid Tumors) | 151 (9.4%) | 139 (14.7%) | 7,93% |
| Chronic Renal Disease (CKD) | 165 (9.1%) | 140 (14.6%) | 0,04% |
| Previous Stroke | 95 (5.3%) | 84 (8.8%) | 0,00% |
| COPD | 87 (4.8%) | 78 (8.2%) | 0,07% |
| Asthma | 83 (4.6%) | 25 (2.6%) | 0,00% |
| Dementia | 38 (2.1%) | 41 (4.3%) | 0,00% |
| Liver Diseases | 43 (2.4%) | 36 (3.8%) | 0,04% |
| Blood Cancer | 33 (2.6%) | 32 (3.8%) | 22,91% |
| Rheumatologic Disease | 54 (3.0%) | 15 (1.6%) | 0,04% |
| Hematological Disease | 21 (2.0%) | 15 (1.7%) | 29,68% |
| HIV/AIDS | 18 (1.0%) | 9 (0.9%) | 0,00% |
| Malnutrition | 9 (0.5%) | 9 (0.9%) | 0,00% |
| CKD on Dialysis | 49 (2.7%) | 39 (4.1%) | 0,07% |
| **Vital Signs on Admission** |  |  |  |
| Temperature | 36.2 (36.0, 37.0) | 36.1 (36.0, 37.0) | 0,22% |
| Heart Rate | 87.0 (78.0, 98.0) | 90.0 (77.0, 101.0) | 0,18% |
| Respiratory Rate | 23.0 (20.0, 27.0) | 24.0 (20.0, 29.0) | 0,47% |
| Systolic Blood Pressure | 124.0 (110.0, 140.0) | 120.0 (103.0, 137.8) | 0,22% |
| Diastolic Blood Pressure | 78.0 (70.0, 84.0) | 72.0 (62.0, 81.0) | 0,22% |
| Perypheral Oxygen Saturation | 94.0 (92.0, 96.0) | 94.0 (90.0, 96.0) | 0,40% |
| **Laboratory values** |  |  |  |
| Lymphocytes (*1.000/mm3), Median (IQR) | 1.0 (0.7, 1.4) | 0.7 (0.5, 1.0) | 2,17% |
| Neutrophils (*1.000/mm3), Median (IQR) | 6.3 (4.3, 9.3) | 9.2 (5.9, 13.3) | 2,21% |
| Platelets (*1000/mm3), Median (IQR) | 234.0 (178.0, 309.0) | 206.0 (147.0, 274.0) | 1,92% |
| C-Reactive Protein (mg/L), Median (IQR) | 100.9 (50.7, 193.4) | 176.1 (93.3, 278.1) | 9,16% |
| Lactic Dehydrogenase (U/L), Median (IQR) | 365.0 (276.0, 488.2) | 527.0 (404.0, 703.0) | 24,68% |
| D-Dimer (ng/dL), Median (IQR) | 1230.0 (691.5, 3112.0) | 2490.0 (1220.0, 9829.0) | 16,32% |
| Urea (mg/dL), Median (IQR) | 38.0 (25.0, 61.0) | 69.0 (44.0, 115.0) | 1,92% |
| Creatinine (mg/dL), Median (IQR) | 0.9 (0.7, 1.3) | 1.4 (0.9, 2.6) | 1,92% |
| **Hospitalization Days,** *Median (IQR)* | 12.0 (7.0, 21.0) | 13.0 (8.0, 23.0) | 0,11% |
| **Admission to ICU,** *Median (IQR)* | 972 (53.8%) | 923 (96.5%) | 0,00% |
| **ICU Days,** *Median (IQR)* | 8.0 (4.0, 15.0) | 11.0 (6.0, 19.0) | 0,00% |
| **Maximum Oxygen Support** |  |  | 2,97% |
| Room Air | 264 (15.2%) | 3 (0.3%) |  |
| Nasal Cannula | 573 (33.0%) | 26 (2.8%) |  |
| Mask | 165 (9.5%) | 17 (1.8%) |  |
| Non-Invasive Ventilation | 87 (5.0%) | 20 (2.1%) |  |
| HFNC | 44 (2.5%) | 9 (1.0%) |  |
| Mechanical Ventilation | 605 (34.8%) | 868 (92.0%) |  |
| **Days on Mechanical Ventilation**, *Median (IQR)* | 8.0 (5.0, 14.0) | 12.0 (7.0, 19.0) | 13,31% |
| **ECMO** | 2 (0.1%) | 9 (0.9%) | 0,00% |
| **Dialysis** | 163 (9.0%) | 475 (49.7%) | 0,00% |
| **Blood Transfusion** | 223 (12.3%) | 338 (35.4%) | 0,00% |
| **Palliative Care** | 10 (0.8%) | 207 (28.0%) | 25,77% |

**S1 Table. Sociodemographic and clinical characteristics by outcome**

**Footnote:** Charlson comorbidity index (CCI); intensive care unit (ICU); high flow nasal cannula (HFNC); mechanical ventilation (MV); extracorporeal membrane oxygenation (ECMO); length of stay (LOS)
