# Supplementary material for: Mortality over time among COVID-19 patients hospitalized during the first surge of the pandemic: A large cohort study
Source: PLoS One. 2022 Sep 28;17(9):e0275212. doi: 10.1371/journal.pone.0275212 (PMC9518866; doi:10.1371/journal.pone.0275212)
Supplement: Attachment — Submitted filename: Response to Reviewers-11-07-2022.docx [file pone.0275212.s002.docx]

Dear Editor,

We are grateful for the opportunity to receive the valuable suggestions from the reviewers. We tried to meet each point of improvement that was raised, leaving the changes made to the text **highlighted with track changes**. We provided an answer below (in red) to each reviewer's commentaries (in blue). We are open to new suggestions and comments that might improve our paper.

Reviewer #1: PONE-D-22-12520 comments

Major comments

The result presentations could be improved by dividing the patients into two cohorts: one in need of intensive care and one that could be treated in the ward. Ideally another group should also be included, patients treated in intermediate care units, but no mentions are given in the manuscript to such units.

Table 1 which is an important source of information should then be divided into three cohorts: All patients, ward patients and ICU patients (both those admitted directly and during hospital stay), and the three phases could be described into this table as well. The mention of ICU patients in several places as now is a bit confusing.

**Answer:** Thank you for the commentary. We agree with the reviewer that patients in need of intensive-care units have different descriptive clinical characteristics (organ dysfunctions, respiratory failure, among others) and this might be interesting to look at. However, we believe that dividing the population into ward and ICU patients would deviate us from the main research question: did mortality change over time, as a consequence of strain? Looking at ICU and ward patients separately might be misleading because during periods of more capacity strain, there was more severe ICU bed scarcity, potentially resulting in moderately ill patients, who would have been admitted to the ICU at one point, remained in the wards at another point, either waiting for a bed or receiving noninvasive support that would typically be administered in the ICU. Other than that, in our opinion Table 1 is very busy as it is, and adding more columns or rows would make it very hard to read.

We did not have intermediate care units in our institution. If the editor feels there are excessive mentions of ICU patients, we are open to revise.

I miss an important information, oxygen saturation at admission (with information of amount of oxygen require), for ICU patients this could be given as the PaO2/FiO2 ratio. This information is important to group patients according to severity at admission, also advocated by WHO guidelines.

**Answer**: Thanks for this commentary. As pointed out, we added the peripheral oxygen saturation, as well as the other vital signs, measured on admission on Tables 1 (by Admission Period) and S1 (Discharged versus Deceased)

**Added to Table 1:**

|  | Total (N=2763) | Early (N=612) | Peak (N=1610) | Late (N=541) |
| --- | --- | --- | --- | --- |
| Peripheral Oxygen Saturation, Median (IQR) | 94.0 (91.0, 96.0) | 94.0 (90.0, 96.0) | 94.0 (91.0, 96.0) | 94.0 (92.0, 97.0) |

I also would like the authors to define what definitions they use for severe acute respiratory syndrome which in addition to age (>18) and COVID-19 test positive.

**Answer:** Thank you for pointing this out. We added the definition of severe acute respiratory syndrome in the methods section.

“probable COVID-19 case was defined as a suspected case (patients presenting with severe acute respiratory syndrome defined as individuals with acute upper respiratory symptoms and presenting with: dyspnea/respiratory discomfort or persistent pressure or pain in the chest or O2 saturation below 95% in room air or cyanosis of the lips or face), with a chest computed tomography or chest-ray suggestive of COVID-19 disease”

You find that mortality was highest during the peak phase. I would like to see a better discussion for this finding. You managed to increase ICU beds 4-fold to 300 beds. With a median hospital LOS of 10 days this capacity over the 4 months (120 days) the hospital could theoretically have 36000 ICU days in the period but actually around 50% were used (18950 days). This means access to an ICU unit were probably not he main problem, do the authors have other explanations like quality of care and (or lack of equipment.

**Answer:** Thank you for the commentary and careful review of the data. We have increased ICU beds capacity to 300 beds, however, we did not have this capacity over the full-time frame as many beds were improvised on surgical rooms, wards and, therefore, during non-peak periods, these beds were not available. Another reason for not being at full capacity during the whole period is the shortage of specialized health professionals (intensivists). We added a sentence explaining this better in the methods section. Lack of ventilators was not a problem in our institution, the main limitation was specialized HCWs. We added a sentence about this to the discussion, as suggested.

“Its ICU capacity was increased by about 4-fold by converting regular wards to ICUs, reaching a total number of 300 ICU beds during the peak of cases period. The Number of available ICU beds was managed as per patient demand during the crisis period.”

Other comments

Page 5, line 99-100, please change the description of income to Upper-Middle Economies, which is according to the most recent World Bank classification.

**Answer:** As suggested, we have changed the description.

Missing important factors for mortality not included in the discussion:

- Change over time:

Jung, C. et al. Differences in mortality in critically ill elderly patients during the second COVID-19 surge in Europe. Crit Care 25, 344 (2021).

- Prognostic indicators found important: ADL and Frailty

Bruno, R. R. et al. The association of the Activities of Daily Living and the outcome of old - intensive care patients suffering from COVID-19. Ann Intensive Care 12, 26 (2022).

Jung, C. et al. The impact of frailty on survival in elderly intensive care patients with COVID-19: the COVIP study. Critical care (London, England) 25, 149 (2021).

**Answer**: Thank you for the comment. We agree frailty is an important risk factor for COVID-19 mortality, and so is baseline performance status. However, we did not collect data on frailty or performance status in our study because we anticipated that our population would not be predominantly composed of elderly patients. Most of the studies accessing the association of frailty and COVID-19 mortality included only elderly patients. Since our cohort included patients 18 years old and over, and the observed median age was 61yo (IQR 48 - 71), we did not include specifically mention frailty in our case report form. We have added a sentence about this to the limitations paragraph.

Reviewer #1:

The authors conducted a retrospective cohort study. Please clarify censor strategies for this cohort.

**Answer**: Thank you for reviewing our article. As suggested, we have better clarified exclusion criteria and censoring in the methods section

“Patients who were admitted in palliative care, patients with nosocomial COVID-19, defined as patients admitted to the hospital complex for other causes, who were infected with SARS-Cov-2 during their hospitalization, and patients transferred to other facilities during their care were excluded from our sample”

and

“No censoring was necessary once all patients admitted during the study period had been discharged when data analysis was performed.”

For the multiple logistic regression, please clarify the methods for variables selection in model. For the multiple regression analysis, the prediction is their objective the model assumptions as well as model performance, a test for the interaction between variables, multicollinearity, a test for the interaction between variables, and goodness-of-fit analysis should be performed and show in the results or supplementary. On the other hand, if the association or casual inference is their aim the confounding factors according to previous knowledge should be included to the model for appropriate effect estimation.

For variable and model selection, please refer to these articles:

I. Heinze G, Wallisch C, Dunkler D. Variable selection - A review and recommendations for the practicing statistician. Biometrical J [Internet]. 2018 May 1;60(3):431–49.

II. VanderWeele TJ. Principles of confounder selection. Eur J Epidemiol [Internet]. 2019 Mar 15;34(3):211–9.

III. Steyerberg EW, Vergouwe Y. Towards better clinical prediction models: seven steps for development and an ABCD for validation. Eur Heart J [Internet]. 2014 Aug 1;35(29):1925–31. Available from: [www.r-project.org](http://www.r-project.org/)

**Answer**: Thank you. The main goal of our analysis was to access if capacity constraints affected mortality rates during peak period, which is inferential. We have chosen the variables to adjust for based on prior knowledge on the topic, and p-values. We agree that the model description can be improved and therefore changes were made to better clarify,

“First, we investigated risk factors associated with overall mortality during hospitalization with a logistic regression model framework: we first conducted a univariate logistic regression analysis, and then fitted a multivariable logistic regression including those independent variables with a resulting odds ratio (OR) with a p-value less than 0.2 from the univariable models. Independent variables included: age group, sex, the Charlson Comorbidity Index (CCI), mechanical ventilation during hospitalization and time of symptoms onset. These variables were chosen to represent both patient intrinsic (age, sex and comorbidities) and disease-severity mortality risk factors (need of mechanical ventilation, days of symptoms onset) and were selected based on prior published literature.

Second, we assessed the impact of the epidemic period on mortality rates. We sub-divided the study timeframe (6-month) in three time-periods according to the stage of the first wave of pandemic in São Paulo: early (March to April); peak (May to June); and late (July to August). The periods represented the different capacity strains the hospital faced (Figure S1). We then modeled both the risk of overall mortality and the probability of a 30-day mortality rate during hospitalization against the period in which the patient was admitted using a logistic regression model. We adjusted for age, sex, the Charlson Comorbidity Index and need for mechanical ventilation, all known risk factors for COVID-19 mortality. ”

Include full details of how the authors handled missing data, outliers and include these in the results section. The author should elaborate about how you were dealing with that.

**Answer**: Thank you for pointing this out. Thanks to an incredible group effort, we had very little missing data. We mention in the methods section about missing data regarding regression analysis (*‘Because of the few missing values, we opted to use a complete-case analysis’*), and now we add a supplemental table with missing percentages per variable to better clarify this issue.

“We used frequency and proportions for categorical variables, and median and interquartile range (IQR) for continuous variables. T-tests, Wilcoxon-rank sum and chi-square tests were performed according to variable distribution and type. Percentage of missing data for each variable is presented on Table S1. Because of the few missing values, we opted to use a complete-case analysis”

Have you tested the associations of the non-linear variables in the regression model, e.g., with splines or a polynomial?

**Answer**: Thank you. We have not run any model to investigate non-linear associations such as splines or polynomials. Although they can provide interesting results, our models included only categorical variables, mostly binary, limiting their applicability. In addition, this was not in our statistical plan prior to analysis.

Please demonstrate flowchart of participants.

**Answer**: Thanks. We have a flowchart of study participants depicted in Figure 1.

Ultimately, the biggest drawback is the lack of practice-changing knowledge.

**Answer**: Thank you for the review. Even though some papers have already addressed capacity constraint and mortality during the pandemic, most of them were conducted in developed countries, and therefore lacked external validity for non-developed countries. In addition, not only the direction of effect, but its magnitude brings knowledge on how different these capacity constraints affected different medical centers, in different countries. Our findings, in light of previous results, can inform organization preparedness plans in scarce-resources situations and potentially help minimize the impact of strain on clinical outcomes in future emergency situations.
